# Role of Mast-Cell-Derived RANKL in Ovariectomy-Induced Bone Loss in Mice

**DOI:** 10.3390/ijms24119135

**Published:** 2023-05-23

**Authors:** Verena Fischer, Jasmin Maria Bülow, Benjamin Thilo Krüger, Deniz Ragipoglu, Anna Vikman, Melanie Haffner-Luntzer, Konstantinos Katsoulis-Dimitriou, Anne Dudeck, Anita Ignatius

**Affiliations:** 1 Institute of Orthopedic Research and Biomechanics, University Medical Center Ulm, 89081 Ulm, Germany; verena.fischer@uni-ulm.de (V.F.);; 2 Institute for Molecular and Clinical Immunology, Otto-von-Guericke University Magdeburg, 39120 Magdeburg, Germany; 3 Health Campus Immunology, Infectiology and Inflammation, Otto-von-Guericke University Magdeburg, 39120 Magdeburg, Germany

**Keywords:** mast cells, RANKL, osteoclasts, post-menopausal osteoporosis, ovariectomy, estrogen, osteoimmunology, mice

## Abstract

Mast cells may contribute to osteoporosis development, because patients with age-related or post-menopausal osteoporosis exhibit more mast cells in the bone marrow, and mastocytosis patients frequently suffer from osteopenia. We previously showed that mast cells crucially regulated osteoclastogenesis and bone loss in ovariectomized, estrogen-depleted mice in a preclinical model for post-menopausal osteoporosis and found that granular mast cell mediators were responsible for these estrogen-dependent effects. However, the role of the key regulator of osteoclastogenesis, namely, receptor activator of NFκB ligand (RANKL), which is secreted by mast cells, in osteoporosis development has, to date, not been defined. Here, we investigated whether mast-cell-derived RANKL participates in ovariectomy (OVX)-induced bone loss by using female mice with a conditional *Rankl* deletion. We found that this deletion in mast cells did not influence physiological bone turnover and failed to protect against OVX-induced bone resorption in vivo, although we demonstrated that RANKL secretion was significantly reduced in estrogen-treated mast cell cultures. Furthermore, *Rankl* deletion in mast cells did not influence the immune phenotype in non-ovariectomized or ovariectomized mice. Therefore, other osteoclastogenic factors released by mast cells might be responsible for the onset of OVX-induced bone loss.

## 1. Introduction

Bone is a dynamic organ that can adapt to mechanical and environmental changes. Under healthy conditions, bone formation by osteoblasts and bone resorption by osteoclasts are in balance to maintain a stable bone mass. However, if bone resorption exceeds bone formation, bone loss occurs and osteoporosis develops, which is associated with skeletal fragility and an increased fracture risk. The most common form is post-menopausal osteoporosis, which develops in females due to a decline in estrogen hormone levels [1]. Across Europe, approximately 26 million women aged over 50 suffer from osteoporosis, with a massive individual and socioeconomic burden [2]. A key mechanism regulating osteoclastic bone resorption is the RANKL/RANK/OPG system. In bone, the receptor activator of the NF-κB ligand (RANKL) is secreted mainly by bone marrow stromal cells, as well as by osteoblasts and osteocytes [3,4,5], and stimulates osteoclast formation after binding to the RANK receptor expressed on pre-osteoclasts [6]. Osteoprotegerin (OPG) was discovered as a soluble decoy receptor for RANKL, thereby reducing osteoclastogenesis [7]. Dysregulations of the RANKL/RANK/OPG signaling system, which arise due to hormonal changes, aging, and environmental and nutritional impacts, can result in the development of bone disorders, including osteoporosis. In this regard, pre-osteoblastic bone marrow stromal cells of post-menopausal women were demonstrated to secrete more RANKL compared to the cells of pre-menopausal women [8]. In addition, an experimental study using the murine ovariectomy (OVX) model of post-menopausal osteoporosis showed that RANKL secreted from osteocytes initiated osteoclastic bone resorption and loss [9]. Post-menopausal osteoporosis is further characterized by a chronic inflammatory status associated with changes in immune cell populations such as neutrophils and T and B lymphocytes [10]. Notably, T and B lymphocytes are also primary sources of RANKL [11,12,13,14], but only B cell-derived RANKL was shown to contribute to bone loss under estrogen-deficient conditions [15,16]. By contrast, the role of RANKL secreted from T cells in this context remains intensively discussed [17]. Interestingly, previous evidence suggested that mast cells, which are resident immune cells of connective tissues that originate in the bone marrow from hematopoietic pluripotent stem cells [18], might also contribute to osteoporosis development. 

Mast cells store in their granules various inflammatory cytokines, chemokines, and growth factors, including interleukin-1β (IL-1β), IL-6, IL-17, tumor necrosis factor (TNF), interferon-γ, transforming growth factor β, C-X-C chemokine motif ligand 1 (CXCL-1), CXCL-8, and CXCL-10, and act as the first line of defense against various antigens [19]. Moreover, mast cell activation and mediator release modulate several physiological and pathological conditions in different settings, including allergy, inflammation, angiogenesis, and tissue homeostasis and repair [20]. Supporting mast cell involvement in osteoporosis, patients with age-related or post-menopausal osteoporosis frequently exhibit an elevated number of mast cells in bone marrow biopsies [21,22,23]. In addition, patients with systemic mastocytosis, which is a condition with abnormally high numbers of mast cells in one or more body organ, including the bone marrow, frequently display a reduced bone mass [24,25,26]. Experimental studies using the rodent OVX model corroborated the results of increased mast cell numbers under osteoporotic conditions and further revealed that mast cells and osteoclasts are frequently co-localized [27,28,29]. Recently, we demonstrated that mast-cell-deficient mice were protected from OVX-induced osteoclastogenesis and bone loss, indicating that mast cells regulate osteoclastogenesis under estrogen-deficient conditions [29]. Moreover, we showed that mast cells trigger disturbed bone fracture healing in osteoporotic mice by regulating fracture-induced inflammation and osteoclastic fracture callus remodeling [30]. Our in vitro experiments further revealed that granular mast cell mediators, particularly histamine, as well as the pro-inflammatory factors Midkine and CXCL-10, exhibited strong osteoclastogenic effects and that their concentrations were significantly diminished in the presence of estrogen [29,30].

Notably, mast cells were also shown to produce RANKL, as reported for the human allergic nasal mucosa, inflamed synovial tissues or atherosclerotic plaques, and for human primary mast cells and different mast cell lines [31,32,33,34]. We recently found that mast-cell-deficient mice exhibited reduced serum RANKL levels and osteoclast numbers after bone fracture under estrogen-deficient conditions [30]. Together, these results imply that mast cells secrete RANKL in various pathological conditions, and this is presumably also dependent on the estrogen status. However, the role of mast-cell-derived RANKL in osteoclastogenesis induction and its contribution to osteoporosis development under estrogen-deficient conditions has, to date, not been investigated.

Therefore, the aim of the present study was to elucidate whether mast-cell-secreted RANKL plays a role in the regulation of physiological bone turnover and the pathology of post-menopausal osteoporosis. For this, we used mice with a specific deletion of *Rankl* in connective-tissue-type mast cells and studied the bone and immune phenotype under physiological conditions and by using the murine OVX model of post-menopausal osteoporosis. 

## 2. Results

### 2.1. Estrogen Decreased Mast Cell RANKL Secretion

**Figure 1:**
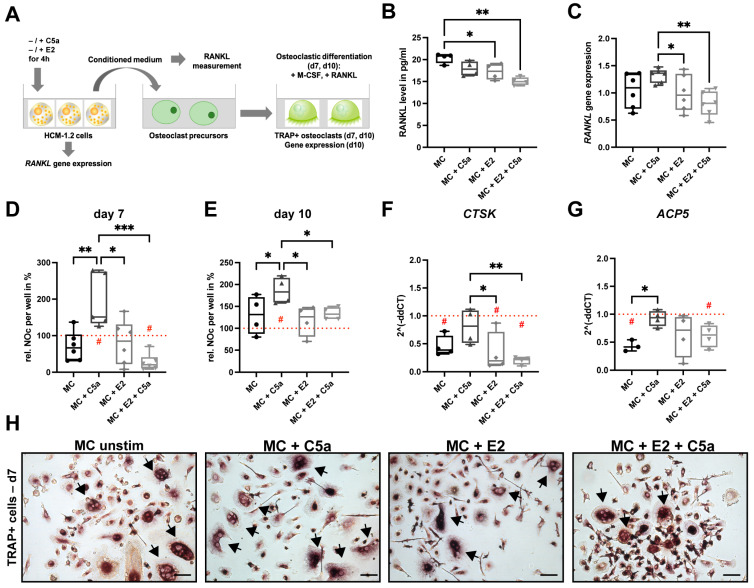
Influence of estrogen (E2) on RANKL secretion in human HMC-1.2 cells. (**A**) Schematic illustration of the in vitro experiment. (**B**) RANKL concentrations in the supernatants of unstimulated mast cells (MC) and C5a-stimulated mast cells (MC + C5a) in the presence and absence of E2, respectively. (**C**) Relative *RANKL* gene expression in HMC-1.2 cells after 4 h of stimulation with/without C5a and E2. (**D**) Relative number of TRAP+ osteoclasts per well in % after 7 days of osteoclastogenic differentiation. (**E**) Relative number of TRAP+ osteoclasts per well in % after 10 days of osteoclastogenic differentiation. Values from osteoclasts treated with the conditioned medium of unstimulated mast cells and mast cells stimulated with C5a, E2, or E2 + C5a were compared with osteoclasts treated with the same conditioned medium without the use of mast cells as controls. Medium controls were set to 100% (red dashed line; # = *p* < 0.05 compared to medium control). (**F**) Gene expression of *CTSK*, and (**G**) *ACP5* in human osteoclast after 10 days of differentiation. Values were normalized to the house keeping gene *GAPDH* and to the same conditioned medium without the use of mast cells as controls (red dashed line; # = *p* < 0.05 compared to the medium control). (**H**) Representative images of TRAP+ stained osteoclasts on day 7 of differentiation (arrows; ≥3 nuclei). * *p* < 0.05, ** *p* < 0.01, *** *p* < 0.001. Scale bar: 50 µm.

To investigate the effect of estrogen on RANKL secretion from mast cells, we used the human HMC-1.2 mast cell line and stimulated these cells with the complement anaphylatoxin C5a to induce rapid mast cell degranulation. C5a was added for 4 h in the presence or absence of estrogen. Subsequently, mast cell supernatants were collected, and RANKL concentrations were determined (Figure 1A).

Both unstimulated and C5a-stimulated mast cells secreted RANKL without showing significant differences in RANKL concentrations between these two groups (Figure 1B). Notably, estrogen treatment significantly decreased RANKL concentrations in supernatants of both unstimulated and C5a-stimulated mast cells (Figure 1B). These results were also confirmed on the gene level, as the relative *RANKL* expression in HMC-1.2 cells was not different between unstimulated and C5a-stimulated mast cells, but the inhibitory effects of estrogen were visible in both groups (Figure 1C). To investigate the effects of mast-cell-secreted factors on osteoclastogenesis, we differentiated primary human monocytes into the osteoclastic lineage in the presence of the supernatants for 7 and 10 days. Supernatants from C5a-stimulated mast cells significantly increased the number of osteoclasts relative to unstimulated mast cells and relative to the medium control after both 7 and 10 days of differentiation (dashed line, set to 100%) (Figure 1D,E,H). Gene expression analysis of osteoclastogenic markers after 10 days confirmed these results, as the *CTSK* gene expression was tendentially (*p* = 0.07) increased, and the *ACP5* gene expression was significantly increased in osteoclasts differentiated in the presence of C5a-activated mast cell supernatants compared to unstimulated mast cell supernatants (Figure 1F,G). Of note, incubation of unstimulated and C5a-stimulated mast cells with estrogen attenuated the strong osteoclastogenic effect of the mast cell supernatants, as indicated by a significantly reduced relative number of osteoclasts compared to the supernatants of C5a-stimulated mast cells which were cultivated without estrogen, as well as compared to the medium control both after 7 and 10 days of differentiation (dashed line, set to 100%) (Figure 1D,E,H). As further confirmation, the *CTSK* gene expression was significantly reduced in the presence of estrogen after 10 days of differentiation (Figure 1F). These results imply that unstimulated and C5a-stimulated mast cells secreted RANKL in an estrogen-dependent manner, whereas only C5a-stimulated mast cells induced osteoclastogenesis.

### 2.2. Rankl Deletion in Mast Cells Did Not Affect Physiological Bone Turnover in Mice

Because our in vitro results showed that mast cells secrete RANKL in an estrogen-dependent manner and regulate osteoclastogenesis, we were interested in whether *Rankl* deletion in mast cells influences bone turnover in vivo. Consequently, we first evaluated the bone phenotype of female and male mice with a specific deletion of *Rankl* in connective-tissue-type mast cells (*Mcpt5-Cre^−^ Rankl*^fl/fl^: control; *Mcpt5-Cre^+^ Rankl*^fl/fl^: *Rankl*-KO (knockout) at different ages. Mast-cell-specific *Rankl* deletion was verified on the protein level by RANKL staining of mast cells (C-KIT) in inguinal lymph nodes (Supplementary Figure S1). 

**Figure 2:**
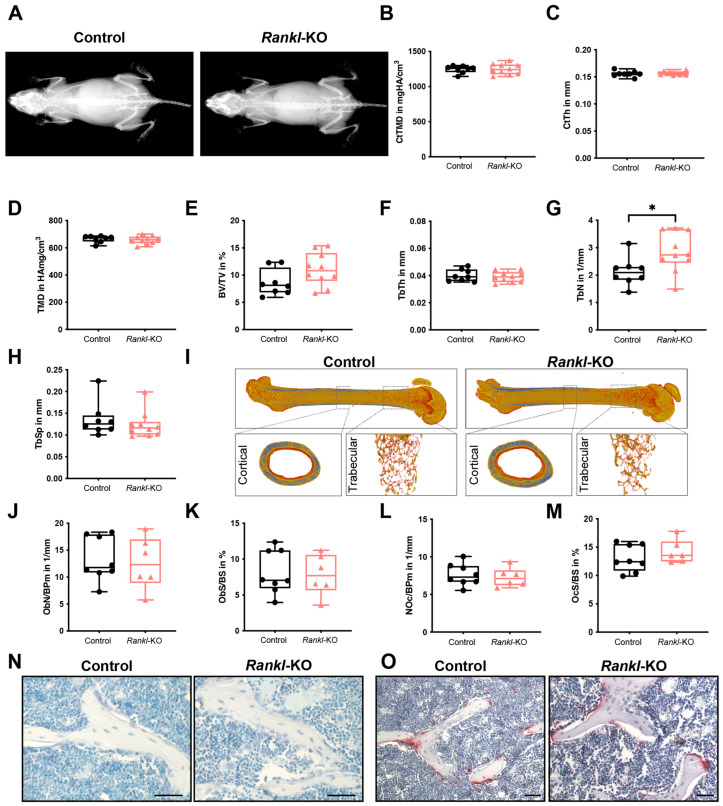
Bone phenotype of the femur of 9–13-week-old female control and *Rankl*-KO mice. (**A**) X-ray images of the entire skeleton. (**B**) Cortical tissue mineral density (CtTMD) and (**C**) cortical thickness (CtTh) at the femur diaphysis. (**D**) Tissue mineral density (TMD), (**E**) bone volume per tissue volume (BV/TV), (**F**) trabecular thickness (TbTh), (**G**) trabecular number (TbN), and (**H**) trabecular separation (TbSp) at the femur condyle. (**I**) Representative three-dimensional µCT reconstructions of the entire bone and cortical and trabecular compartments. (**J**) Number of osteoblasts per bone perimeter (NOb/BPm), (**K**) osteoblast surface per bone surface (ObS/BS), (**L**) number of osteoclasts per bone perimeter (NOc/BPm), and (**M**) osteoclast surface per bone surface (OcS/BS) at the trabecular part of the femur. (**N**) Representative images of Toluidine blue-stained osteoblasts in the trabecular part of the femur. Scale bar: 50 µm. (**O**) Representative images of TRAP+ stained osteoclast in the trabecular part of the femur. * *p* < 0.05. Scale bar: 50 µm.

When aged 9–13 weeks, female *Rankl*-KO mice did not display any macroscopic skeletal abnormalities based on X-ray analysis compared to controls (Figure 2A). Furthermore, there were no differences in cortical tissue mineral density (CtTMD) or cortical thickness (CtTh) of the femurs of the control and *Rankl*-KO mice (Figure 2B,C). Micro-computed tomography (µCT) analysis of the trabecular bone compartment of the femur further revealed no differences between female control and *Rankl*-KO mice in TMD (Figure 2D), bone volume fraction (BV/TV) (Figure 2E), trabecular thickness (TbTh) (Figure 2F), or trabecular separation (TbSp) (Figure 2H). Only the trabecular number (TbN) was slightly but significantly increased in *Rankl*-KO mice compared to controls (Figure 2G) but with no significant consequences on the bone mass or structural parameters. In support of these findings, there were no significant differences between control and *Rankl*-KO mice in the number of osteoblasts per bone perimeter (NOb/BPm) and in the osteoblast surface per bone surface (ObS/BS), as well as in the number of osteoclasts per bone perimeter (NOc/BPm) and the osteoclast surface per bone surface (OcS/BS) (Figure 2J–O).

Female control and *Rankl*-KO mice aged 21–24 weeks also did not exhibit major alterations in the cortical or trabecular bone compartments, nor in cellular bone parameters (Table 1). Only the CtTh of *Rankl*-KO mice was slightly but significantly reduced compared to control mice (Table 1).

*Rankl* deletion in mast cells also did not affect the bone phenotype of male mice aged 9–13 or 21–24 weeks (Supplemental Table S1). An age-dependent increase in TbTh was recognized in both male control and *Rankl*-KO mice (Supplemental Table S1).

In summary, *Rankl* deletion in mast cells did not alter the bone phenotype of female or male mice at either age under physiological conditions. These results suggested that mast-cell-derived RANKL does not play an essential role in physiological bone turnover.

### 2.3. Rankl Deletion in Mast Cells Did Not Protect from OVX-Induced Bone Loss in Mice

**Figure 3:**
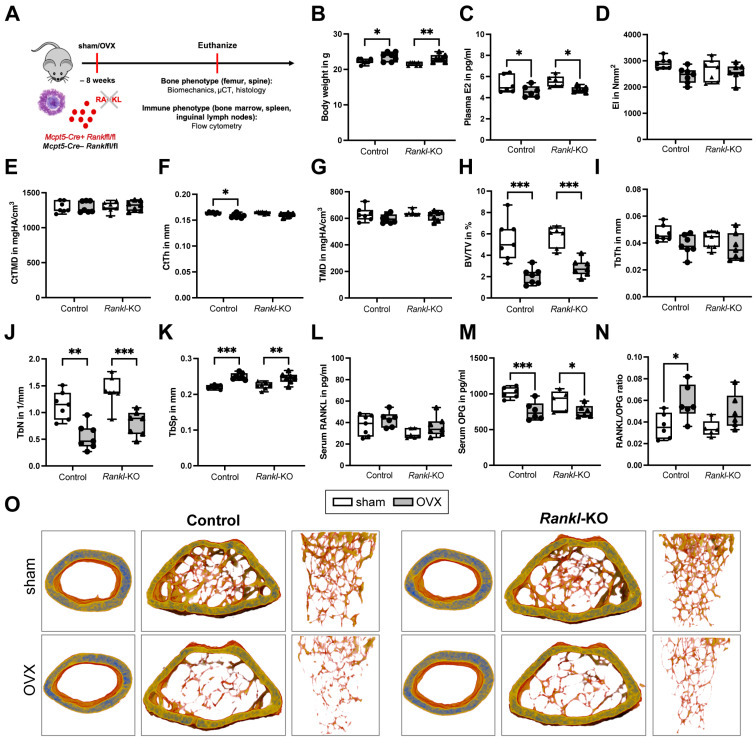
Bone phenotype of the femur of control and *Rankl*-KO sham and ovariectomized mice. (**A**) Schematic illustration of the in vivo study. (**B**) Body weight and (**C**) plasma estrogen levels after sham or ovariectomy (OVX) surgery. (**D**) Bending stiffness (EI) of the femur. (**E**) Cortical tissue mineral density (CtTMD) and (**F**) cortical thickness (CtTh) at the femur diaphysis. (**G**) Tissue mineral density (TMD), (**H**) bone volume per tissue volume (BV/TV), (**I**) trabecular thickness (TbTh), (**J**) trabecular number (TbN), and (**K**) trabecular separation (TbSp) at the trabecular part of the femur condyle. (**L**) Serum RANKL levels, (**M**) serum OPG levels, and (**N**) RANKL/OPG ratio after sham or OVX surgery. (**O**) Representative three-dimensional µCT reconstructions of the cortical and trabecular compartments of the femur. * *p* < 0.05, ** *p* < 0.01, *** *p* < 0.001.

To investigate the role of mast-cell-derived RANKL in OVX-induced bone loss, female mice aged 12 weeks underwent OVX or sham surgery, and the bone phenotype was analyzed (Figure 3A). In both control and *Rankl*-KO mice, the body weight was significantly increased (Figure 3B) after OVX, whereas plasma estrogen levels were significantly reduced (Figure 3C), thus demonstrating the success of the OVX surgery and estrogen decline. The bending stiffness (EI) (Figure 3D) and the CtTMD (Figure 3E) of the femurs were not significantly changed in both control and *Rankl*-KO mice after OVX. However, control mice displayed a significantly reduced femoral CtTh after OVX compared to sham mice (Figure 3F). The OVX did not significantly affect the trabecular TMD of both control and *Rankl*-KO mice (Figure 3G), but the trabecular bone mass in the femur was significantly reduced compared to sham mice in both groups, as indicated by a significantly reduced BV/TV (Figure 3H) and TbN (Figure 3J), as well as a significantly increased TbSp (Figure 3K). There were no differences in TbTh between the groups (Figure 3I). Representative three-dimensional µCT reconstructions of the cortical and trabecular compartment of the femurs clearly demonstrated the reduced bone mass in both control and *Rankl*-KO mice after OVX (Figure 3O).

Analysis of the bone turnover markers in the serum further confirmed these results. Following OVX, serum RANKL levels did not differ significantly compared to the corresponding sham groups (Figure 3L). However, sham *Rankl*-KO mice displayed tendentially reduced serum RANKL levels (*p* = 0.09) compared to sham control mice (Figure 3L), thus suggesting that mast-cell-secreted RANKL enters the circulation but is clearly not significantly affected by the OVX. Serum OPG levels were significantly reduced both in control and *Rankl*-KO mice after OVX (Figure 3M). Furthermore, the RANKL/OPG ratio was significantly increased in control mice and, by trend, increased (*p* = 0.08) in *Rankl*-KO mice after OVX (Figure 3N), thus indicating an increased bone resorptive status after OVX.

Confirming the trabecular bone phenotype of the femur, ovariectomized control and *Rankl*-KO mice displayed a significantly reduced trabecular bone mass in the spine, as demonstrated by a significantly decreased BV/TV, TbTh, and TbN (Table 2). TMD and TbSp were only affected in control mice after OVX (Table 2).

**Figure 4:**
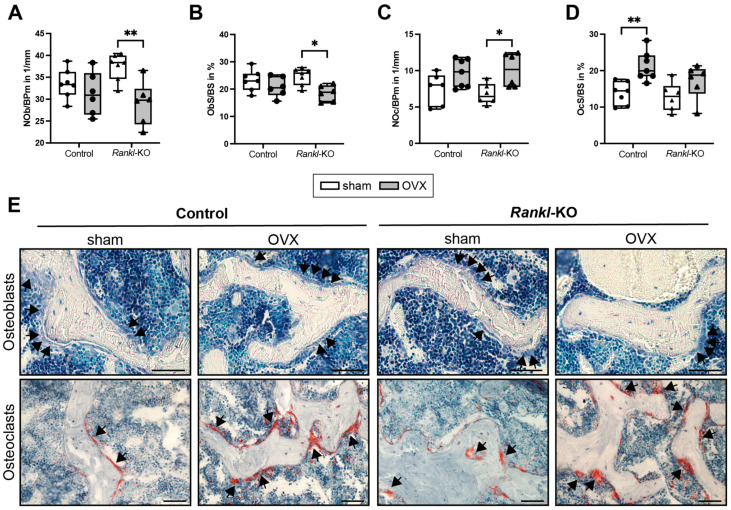
Cellular parameters in the trabecular compartment of the spine of control and *Rankl*-KO sham and ovariectomized mice. (**A**) Number of osteoblasts per bone perimeter (NOb/BPm), (**B**) osteoblast surface per bone surface (ObS/BS), (**C**) number of osteoclasts per bone perimeter (NOc/BPm), and (**D**) osteoclast surface per bone surface (OcS/BS). (**E**) Representative images of Toluidine blue-stained osteoblasts (arrows) and TRAP+ stained osteoclasts (arrows) in the spines of the mice. * *p* < 0.05, ** *p* < 0.01. Scale bar: 50 µm. OVX: ovariectomy.

The histological analysis of the cellular parameters in the spine revealed no changes in osteoblast parameters (NOb/BPm or ObS/BS) in control mice after OVX (Figure 4A,B,E). By contrast, OVX control mice displayed a significantly increased OcS/BS compared to sham mice (Figure 4D,E), which was in agreement with the reduced OPG levels and increased RANKL/OPG ratio measured in the serum of these mice (Figure 3M,N), and indicated an increased osteoclast resorptive activity. Furthermore, *Rankl*-KO mice displayed a significantly reduced NOb/BS and ObS/BS compared to sham mice (Figure 4A,B,E). In addition, the NOc/BPm was significantly increased in *Rankl*-KO mice after OVX (Figure 4C,E), which was supported by the reduced OPG levels and increased RANKL/OPG ratio found in the serum of these mice (Figure 3M,N). In conclusion, the reduced bone mass in both control and *Rankl*-KO mice after OVX is the result of an imbalance in bone formation and its resorption.

In summary, these results showed that *Rankl*-KO mice were not protected from OVX-induced bone loss, suggesting that mast-cell-derived RANKL is not the key mast cell regulator of osteoclastogenesis or bone loss under estrogen-deficient conditions.

### 2.4. Rankl Deletion in Mast Cells Only Marginally Affected the Immune Phenotype after OVX 

**Figure 5:**
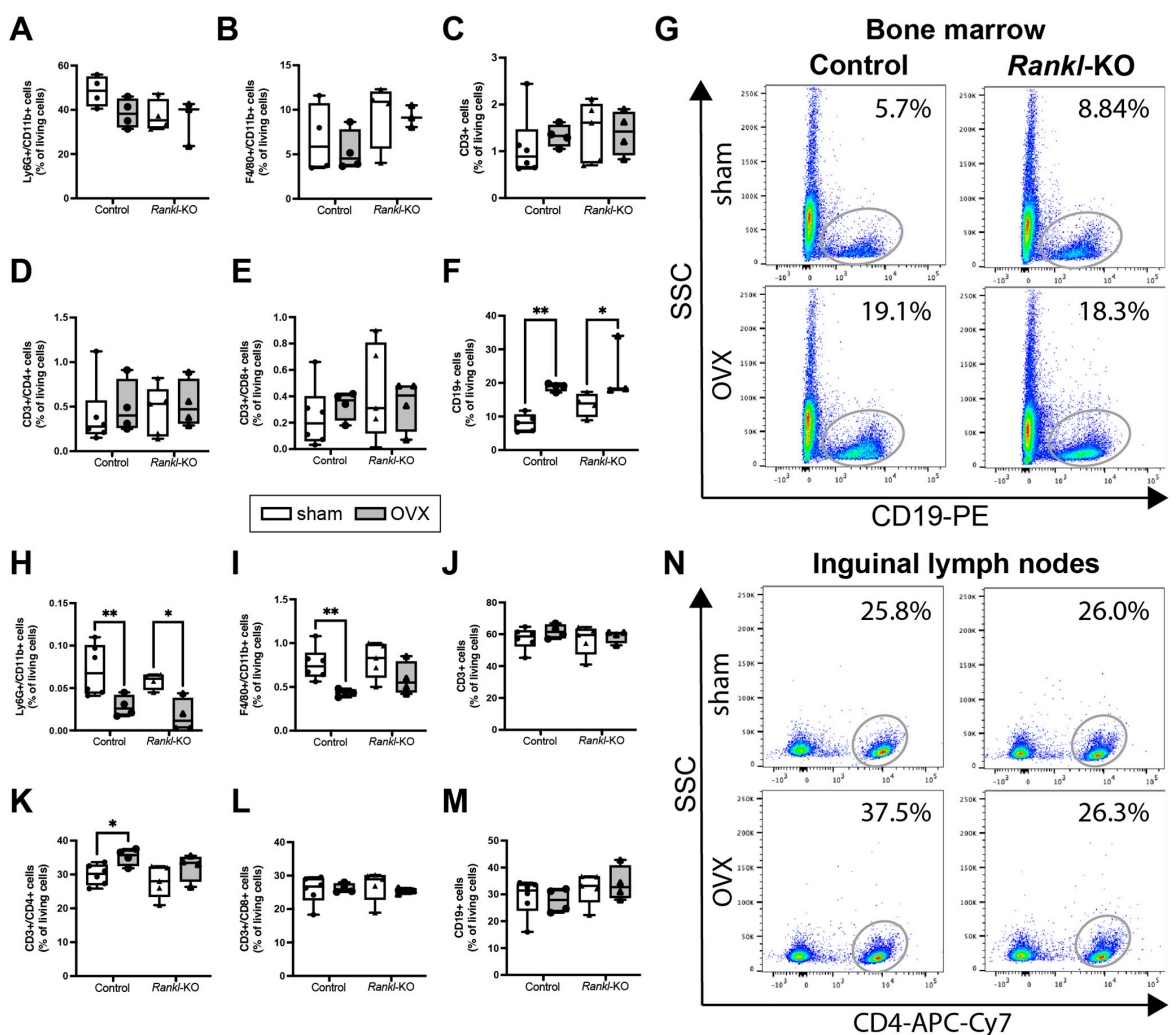
Immune cell populations in the bone marrow and inguinal lymph nodes of control and *Rankl*-KO sham and ovariectomized mice. (**A**) Neutrophils (Ly6G+/CD11b+), (**B**) macrophages (F4/80+/CD11b+), (**C**) T lymphocytes (CD3+), (**D**) T helper cells (CD3+/CD4+), (**E**) T cytotoxic cells (CD3+/CD8+), and (**F**) B lymphocytes (CD19+) in the bone marrow. (**G**) Representative dot plots of CD19+ B lymphocytes in the bone marrow of control and *Rankl*-KO sham and ovariectomized mice. (**H**) Neutrophils (Ly6G+/CD11b+), (**I**) macrophages (F4/80+/CD11b+), (**J**) T lymphocytes (CD3+), (**K**) T helper cells (CD3+/CD4+), (**L**) T cytotoxic cells (CD3+/CD8+), and (**M**) B lymphocytes (CD19+) in inguinal lymph nodes. (**N**) Representative dot plots of CD3+/CD4+ T helper cells in the inguinal lymph nodes of control and *Rankl*-KO sham and ovariectomized mice. OVX: ovariectomy. SSC: sideward scatter. * *p* < 0.05, ** *p* < 0.01.

Because mast cells are important sensor and effector cells of the innate immune system [20,35], and because RANKL has many functions in the immune system [36], we investigated whether *Rankl* deletion in mast cells also influenced the immune phenotype of female sham and ovariectomized mice (Figure 3A). Flow cytometric analysis of the bone marrow, inguinal lymph nodes, and spleen revealed that *Rankl* deletion in mast cells did not affect the proportion of innate and adaptive immune cells under physiological conditions (Figure 5, Supplemental Table S2), because there were no significant changes in macrophages, neutrophils, T lymphocytes, T helper cells, T cytotoxic cells, or B cells between control and *Rankl*-KO mice of the sham groups. Following OVX, both control and *Rankl*-KO mice displayed an increased percentage of B cells in the bone marrow compared to the respective sham groups (Figure 5F,G).

In inguinal lymph nodes, the percentage of neutrophils was significantly reduced in both control and *Rankl*-KO mice after OVX (Figure 5H). By contrast, the percentage of macrophages was significantly reduced (Figure 5I) and the percentage of T helper cells was significantly increased (Figure 5K,N) only in the inguinal lymph nodes of control mice after OVX, but not in *Rankl*-KO mice after OVX. Furthermore, neutrophils were significantly reduced in the spleen of control mice after OVX (Supplemental Table S2).

These data indicated that *Rankl* deletion in mast cells did not influence the immune phenotype under physiological conditions and only marginally affected neutrophils, macrophages, and T helper cells after OVX but clearly with no significant consequences on the bone phenotype of these mice.

## 3. Discussion

Osteoporosis is globally the most common skeletal disease and is caused by multifactorial conditions, including changes in the hormonal, environmental, and nutritional status [1]. In this study, we demonstrated that mast-cell-derived RANKL, the key factor regulating osteoclast formation, is not involved in the pathology of post-menopausal osteoporosis when using mice with a specific deletion of *Rankl* in mast cells.

In recent years, evidence suggested that mast cells regulate bone turnover under pathological conditions, such as post-menopausal osteoporosis [21,23]. In support of this, we and others showed that more mast cells were present in the bone marrow of ovariectomized mice, thereby stimulating osteoclastic bone resorption [27,29]. The strong osteoclastogenic effects were mediated mainly by granular mast cell mediators and were diminished in the presence of estrogen [29,30]. However, the role of RANKL, which has been shown to be released by mast cells of various tissues [31,33,34], in this context has, to our best knowledge, not been investigated until now.

Therefore, by using the human HMC-1.2 cell line, we first investigated in vitro the effects of mast cell stimulation on the gene expression of *RANKL* and its protein secretion dependent on estrogen. We used the complement anaphylatoxin C5a as a mast cell stimulus, because it triggers a rapid and massive release of granular mast cell mediators [37,38]. Furthermore, complement factors were shown to be increasingly expressed in the blood and locally in bones of post-menopausal, osteoporotic females [39,40]. Moreover, we recently revealed that complement signaling in osteoblasts is significantly involved in OVX-induced bone loss in mice [41]. Here, we found that both unstimulated and C5a-stimulated mast cells expressed and secreted RANKL after 4 h on the gene and protein level, with no differences between the groups. Notably, the presence of estrogen significantly reduced *RANKL* gene expression and RANKL levels in both groups. The inhibitory effects of estrogen and estrogen receptor signaling on RANKL secretion were already described for cells of the osteoblastic lineage [42,43]. Our results indicated that mast-cell-RANKL secretion is also strongly dependent on estrogen; however, it is likely independent of the C5a stimulation, at least after 4 h of stimulation with C5a. Kim et al. showed that stimulation of the human mast cell line LUVA with IL-33 for 24 h significantly increased RANKL production [33]. By contrast, Ng et al. did not detect secreted RANKL in the culture medium of unstimulated or stimulated mast cells; however, the authors used primary human mast cells, stimulating these cells with anti-IgE or substance P for 24 h [32], which might explain the differences in the observed results. Accordingly, mast-cell-RANKL secretion appears to strongly depend on the activating stimuli, timing, and cell type used; but limiting our study, we did not investigate different stimuli or further time points. Subsequently, we were interested in whether mast-cell-released mediators stimulate osteoclastogenesis, which is why we differentiated primary human monocytes into osteoclasts in the presence and absence of the collected mast cell supernatants. Although we found that both unstimulated and C5a-stimulated mast cells secreted RANKL, only the supernatants of C5a-stimulated mast cells strongly induced osteoclast formation after 7 and 10 days of differentiation and increased the gene expression of osteoclastogenic markers after 10 days of differentiation, which was significantly diminished in the presence of estrogen and is supported by our previous studies [29,30]. Nevertheless, limiting our study, we did not investigate the resorptive activity of human osteoclasts under the various treatment conditions by using a pit resorption assay. However, our previous study revealed that not only the number of osteoclasts was increased in the presence of C5a-activated mast cell supernatants but also their resorptive capacity [29]. Thus, we assume that in our present setting, the resorptive activity of osteoclasts that were differentiated in the presence of C5a-activated mast cell supernatants might be affected accordingly. Our recent findings imply that mast-cell-secreted RANKL was not the major driver of osteoclastogenesis in this setting. We previously reported that histamine and the pro-inflammatory cytokine Midkine may act as key osteoclastogenic factors secreted by mast cells under estrogen-deficient conditions [29,30]; however, in vivo confirmation remains lacking. Others recently showed that direct cell–cell contact by membrane-bound RANKL is the main driver of mast-cell-induced osteoclast formation [32,33]. As a limitation of our study, we did not distinguish between the effects of mast-cell-secreted and membrane-bound RANKL by using direct co-culture or trans-well systems. Furthermore, to the best of our knowledge, we are not aware of the existence of various isoforms of RANKL differentially expressed by mast cells that may play similar roles. In conclusion, our in vitro results showed that mast cells secrete RANKL independently of the C5a stimulation but strongly dependently on estrogen. Thus, and as the in vitro system only partially mimics the in vivo situation, our main focus was to investigate the role of mast-cell-derived RANKL in OVX-induced bone loss in vivo.

For that, we used mice with a specific *Rankl* deletion in connective-tissue-type mast cells (*Mcpt5-Cre Rankl*^fl/fl^) and first determined the effects on physiological bone turnover. The lack of RANKL staining in the mast cells of *Rankl*-KO mice confirmed its successful deletion. Furthermore, serum RANKL levels were decreased in female *Rankl*-KO mice compared to controls, indicating that mast-cell-derived RANKL contributes to systemic RANKL concentrations. In this study, we demonstrated that *Rankl*-KO mice did not develop an altered cortical or trabecular bone phenotype in the femur at any age or in either sex. Only the cortical thickness and trabecular number were significantly changed in female *Rankl*-KO mice compared to controls, though without affecting the overall bone structure or mass. Furthermore, in males, a similar age-dependent increase in trabecular thickness was observed in both these mouse types. These results suggested that mast-cell-derived RANKL is redundant in physiological bone turnover. Corroborating these results, mast-cell-deficient Mcpt5-Cre R-DTA mice similarly did not display changes in physiological bone turnover [29]. By contrast, Kit-dependent mast-cell-deficient mice exhibited a high-bone-turnover osteopenia [44], but the cell surface receptor Kit is also expressed on other immune cells and on bone cells [45,46]. Notably, we did not detect changes in immune cell populations between female control and *Rankl*-KO mice, thus indicating that mast cell *Rankl* deletion did not affect the immune phenotype under physiological conditions. However, mast-cell-derived RANKL might exert crucial effects in bone resorption when the system is challenged. Supporting this proposal, increased mast cell numbers and elevated RANKL levels were found in the synovial tissues of patients with rheumatoid arthritis or osteoarthritis, thereby contributing to inflammatory bone erosions [33,47,48]. Moreover, in arteriosclerotic plaques, mast cell RANKL expression was associated with coronary artery lesions [31]. A positive correlation between mast cells and blood RANKL levels was observed in patients with multiple-myeloma-related bone osteolysis [49]. In rats, mast cell depletion improved alveolar bone loss in periodontal disease associated with a reduced RANKL expression [50]. Furthermore, serum RANKL levels and osteoclast numbers were significantly reduced in mast-cell-deficient mice after fracture both under normal and estrogen-deficient conditions [29,30]. These findings strongly supported an involvement of mast-cell-derived RANKL in various pathological and inflammatory conditions. 

Post-menopausal osteoporosis is also regarded as a chronic inflammatory disease with increased circulatory levels of inflammatory cytokines such as TNF, IL-6, or IL-1β and changes in immune cell populations [10]. Therefore, we examined both the bone and immune phenotype of *Rankl*-KO mice after OVX. Verifying the success of the OVX surgery, we observed an increased body weight in both control and *Rankl*-KO mice, accompanied by a decline in blood estrogen levels, which was in agreement with previously published studies [51,52,53]. However, in the present study, we found that the specific deletion of *Rankl* in mast cells did not protect from the OVX-induced increase in osteoclastogenesis or bone loss. Both control and *Rankl*-KO mice displayed a reduced trabecular bone mass in the femur and lumbar spine after OVX, which resulted from reduced osteoblast and increased osteoclast parameters that were associated with an increased RANKL/OPG ratio. In support of these in vivo results, only the supernatants of C5a-stimulated mast cells induced osteoclast formation in vitro, although both unstimulated and C5a-stimulated mast cells expressed and secreted RANKL into the culture medium. Therefore, our results suggested that mast-cell-derived RANKL is not the key regulator of the osteoclast-stimulating effects of mast cells under estrogen-deficient conditions. However, as a limitation of our study, we could not check the expression level of *Rankl* in primary murine mast cells isolated from the bone marrow, as a complex digestion protocol is needed for cell isolation, which negatively affects their cell surface marker expression. Furthermore, as it is known that mast cell maturation and mediator content strongly depend on the growth factors and microenvironment provided by the tissue [20], *RANKL* expression in mast cells may vary between different tissues. Nevertheless, in further support of our in vivo findings, we did not detect major changes in immune cell populations between control and *Rankl*-KO mice after OVX. Both groups exhibited an increased number of B cells in the bone marrow after OVX, which was in agreement with previous studies in ovariectomized mice [54]. Notably, B cells were shown to contribute to bone resorption under estrogen-deficient conditions by secreting granulocyte colony-stimulating factor and RANKL [55,56]. Furthermore, neutrophil numbers were significantly reduced in the inguinal lymph nodes of both control and *Rankl*-KO mice after OVX, which was in contrast to clinical data showing an increased neutrophil-to-lymphocyte ratio in post-menopausal females [57]. However, neutrophil numbers may vary between the circulation and specific organs and might strongly depend on the severity of estrogen deficiency. Interestingly, only control mice displayed significantly reduced macrophage numbers in inguinal lymph nodes after OVX. A previous study revealed that OVX significantly affected M1 and M2 polarization under inflammatory conditions [58]. Interestingly, RANKL was shown to induce a switch from M2 to M1 macrophages [59], and M2 macrophages were reported to differentiate into mature osteoclasts in the presence of RANKL only under estrogen-deficient conditions [60]. Because we did not detect differences in macrophages in *Rankl*-KO mice after OVX, it might be that mast-cell-derived RANKL is involved in macrophage polarization; however, limiting our study, we did not further distinguish between macrophage subtypes. Moreover, CD4+ T helper cells were significantly increased in inguinal lymph nodes of control mice only after OVX, which was in agreement with clinical data showing increased circulatory T cells in post-menopausal females [61]. In addition, we previously showed that ovariectomized mice display more CD4+ T helper cells in the bone marrow [62]. It was already shown that RANKL ensures T helper cell polarization [63]. Because CD4+ T helper cells were not changed in *Rankl*-KO mice after OVX, this might indicate that mast-cell-derived RANKL affects T cell differentiation; however, we did not distinguish further between different T helper cell subsets. Nonetheless, the observed changes in immune cell populations clearly did not interfere with the OVX-induced bone loss observed in both control and *Rankl*-KO mice. We also measured inflammatory mediators in the blood of sham and ovariectomized mice, but only a few mediators were detectable, and there were no significant differences between all mice. Nevertheless, mast-cell-released RANKL could play a more critical role in models with higher inflammation, such as during fracture healing, in sepsis, or after severe trauma, which is further supported by its pivotal role during arteriosclerosis or arthritis [31,33].

In summary, our in vitro and in vivo results suggested that mast-cell-derived RANKL is not involved in the pathology of post-menopausal osteoporosis, which are crucial results, as RANKL is the master regulator of osteoclastogenesis. Recently, we observed significantly reduced serum levels of the pro-inflammatory and osteoclast stimulating factors Midkine and CXCL-10 in mast-cell-deficient mice after fracture under estrogen-deficient conditions [30]. We further showed that human HMC-1.2 cells secreted both Midkine and CXCL-10 upon C5a-stimulation, the levels of which were diminished in the presence of estrogen. Moreover, adding an inhibitory Midkine antibody to the mast cell supernatants strongly reduced their osteoclastogenic potential [30]. Therefore, possible osteoclast-stimulating factors released from mast cells that could be involved in osteoporosis development might be Midkine and/or CXCL-10, which needs to be verified in future studies. Beyond that, unbiased proteome analysis of the mast-cell-secretome should be performed in future studies, in order to identify mast cell mediators that might play a role in osteoclastogenesis and bone loss that is dependent on estrogen. 

## 4. Materials and Methods

### 4.1. In Vitro Experiments with HMC-1.2 Cells

To analyze the effects of estrogen on mast cell RANKL secretion, the HMC-1.2 human mast cell line (Merck Millipore, Burlington, MA, USA) was used. Cells were cultured and expanded in Isove medium (Merck Millipore) that was supplemented with 1.2 mM β-thioglycerol (Sigma-Aldrich, St. Louis, MO, USA), 10% fetal bovine serum (Merck Millipore), and 1% penicillin/streptomycin (Gibco, Thermo Fisher Scientific, Waltham, MA, USA). Mast cell degranulation of 2 × 10^6^ cells/mL was induced via stimulation with the anaphylatoxin C5a (rapid and massive degranulation of stored mediators) (R&D systems, Minneapolis, MN, USA) for 4 h in the presence or absence of estrogen, followed by the collection of mast cell supernatants. RANKL concentrations in mast cell supernatants were determined by using a customized human TNFSF11 (antibodies-online, Aachen, Germany) enzyme-linked immunosorbent assay (ELISA) kit according to the manufacturer’s protocol.

To analyze the effects of mast cell supernatants on osteoclastogenesis, primary human monocytes were freshly isolated from the peripheral blood of healthy donors, as described previously [30], after written informed consent was obtained according to the terms of the ethics committee of Ulm University, Germany. Freshly isolated monocytes were seeded on 96-well plates and differentiated into osteoclast-like tartrate-resistant acid phosphatase (TRAP)+ multi-nucleated cells by using minimum essential medium α (Gibco, Thermo Fisher Scientific), 10% fetal calf serum (Gibco, Thermo Fisher Scientific), 1% L-glutamine (Gibco, Thermo Fisher Scientific), 1% penicillin/streptomycin (Gibco, Thermo Fisher Scientific), recombinant human (rh) RANKL (25 ng/mL, R&D systems), and rhM-CSF (10 ng/mL, Merck Millipore). In total, 500,000 cells/cm^2^ were seeded and stimulated with the collected mast cell supernatants (1:20 *v/v* in medium) at 37 °C with 5% CO_2_ under a humid atmosphere. Media containing only C5a or estrogen were used as controls. At day 7 and day 10 after stimulation, the formation of TRAP+ multi-nucleated cells (≥3 nuclei) was investigated via positive TRAP staining (Acid Phosphatase Leucocyte Kit, Sigma-Aldrich) and visualized via light microscopy (Leica DMI6000 B, Leica, Wetzlar, Germany). For gene expression analysis, we isolated the RNA from HMC-1.2 cells (after 4 h of stimulation) and from differentiated human osteoclasts (day 10) as described previously by using the RNeasy Micro Kit (Qiagen, Hilden, Germany) [29]. Subsequently, by using the Omniscript Reverse Transcriptase Kit (Qiagen), 1 µg of RNA was transcribed into cDNA. We used the BrilliantSybr Green QPCR MasterMix Kit (Stratagene, Amsterdam, Netherlands) to perform qPCR as described previously [29]. Gene expression of *GAPDH* (forward: 5′ GAA GGT GGT CGG AGT AGT C 3′; reverse: 5′ GAA GAT GGT GAT GGG ATT TC 3′); *TNFSF11* (forward: 5′ GCC ACC AAA GAA TTG CAG ATG ATG G 3′; reverse: 5′ TCT GCA TTT TCA TGG AGT CTC A 3′); *CTSK* (forward: 5′ TTC CAT CAG CAG GAT GTG GG 3′; reverse: 5′ TCC CAG TGG GTG TCC AGT AT 3′); and *ACP5* (forward: 5′ CCG CCA GGA GTG CGA t 3′; reverse: 5′ GAG TCC CTT CAG TCC CTG C 3′) was analyzed by using the delta–delta CT method. Values were normalized to the house keeping gene *GAPDH* and to the respective medium controls. 

### 4.2. In Vivo Experimental Mouse Model

*Mcpt5-Cre Rankl*^fl/fl^ mice (kindly provided by Anne Dudeck, Otto-von-Guericke University Magdeburg, Germany) were housed in the Animal Facility of Ulm University (Ulm, Germany) in groups of two to five mice under standard rodent conditions with a 12 h light, 12 h dark rhythm, and water and standard mouse feed (Sniff R/M-H, V1525-300, Ssniff, Soest, Germany) ad libitum. The mice were on a C57BL/6 background. Littermates were used as controls. All animal experiments were in compliance with international guidelines for the care and use of laboratory animals (ARRIVE guidelines and EU Directive 2010/63/EU for animal experiments) with the approval of the Local Ethical Committee (no. 1531, o.135-10, Regierungspraesidium Tuebingen, Germany). For genotyping, ear biopsies were used, and genomic DNA was extracted by using the DirectPCR Lysis Reagent (Viagen Biotech, Los Angeles, CA, USA) including a proteinase K (1:100, Bioline, London, UK) digestion step. PCR was performed as previously described by using the following primers: *Mcpt5*-Ex1 DO3: 5′ TGA GAA GGG CTA TGA GTC CCA 3′; *Mcpt5*-Cre UP: 5′ ACA GTG GTA TTC CCG GGG AGT GT 3′; *M5/M6*-Cre DO: 5′ GTC AGT GCG TTC AAA GGC CA 3′; *Tnfrsf11*_15250_for: 5′ GGC ACA TGG TCA CTT GGT AG 3′; *Tnfrsf11*_15251_rev: 5′ AGC TTT TAG AAT GCC AAT AAT TAA A 3′ (Supplemental Figure S1).

Successful *Rankl* deletion in mast cells was confirmed on the protein level by immunofluorescent staining for RANKL and C-KIT (mast cells) in inguinal lymph nodes of control (*Mcpt5-Cre^−^ Rankl*^fl/fl^) and *Rankl*-KO (*Mcpt5-Cre^+^ Rankl*^fl/fl^) mice that were housed in the Animal Facility of Otto-von-Guericke University Magdeburg (Magdeburg, Germany) with the approval of the Local Ethical Committee (no. 42502-2-1416UniMD, Landesverwaltungsamt Sachsen-Anhalt, Germany). For that, inguinal lymph nodes from the back were isolated and directly frozen in Tissue-Tek^®^ O.C.T. Compound (Sakura Finetek Europe B.V., Alphen aan den Rijn, The Netherlands). Cryo-sections of 10 µm were prepared and adhered to silane-coated cover slides. Subsequently, the slides were fixed with 2% *v/v* paraformaldehyde, permeabilized with 0.2% *v/v* Triton X-100, and blocked with 1% *w/v* bovine serum albumin in 1× PBS. Slides were stained with α-RANKL antibody (Clone IK22/5, Biolegend, San Diego, CA, USA) and C-KIT antibody (CD117 clone ACK2, Biolegend) and analyzed by using an inverted wide-field fluorescence microscope (Leica DMI600, Leica) (Supplemental Figure S1).

### 4.3. Bone Phenotyping

Male and female control (*Mcpt5-Cre^−^ Rankl*^fl/fl^) and *Rankl*-KO (*Mcpt5-Cre^+^ Rankl*^fl/fl^) mice aged 9–13 weeks and 21–24 weeks were used to investigate whether mast-cell-specific *Rankl* deletion affected physiological bone turnover. For that, mice were euthanized by using an isoflurane overdose and terminal intracardiac blood withdrawal. The skeletons of all mice were analyzed via X-ray imaging (35 kV, 11 s, Faxitron, Hologic Inc., Marlborough, MA, USA), µCT, and histomorphometry, as described in detail below.

### 4.4. Ovariectomy

Female control (*Mcpt5-Cre^−^ Rankl*^fl/fl^) and *Rankl*-KO (*Mcpt5-Cre^+^ Rankl*^fl/fl^) mice aged 12 weeks were randomly assigned to the different treatment groups that were either subjected to bilateral OVX to mimic estrogen-decline and post-menopausal osteoporosis in humans or sham-operated as described previously [64]. Directly after sham/OVX surgery, standard mouse feed was changed to a phytoestrogen-free diet (Ssniff). At 8 weeks post-surgery, all mice were euthanized by using an isoflurane overdose and terminal intracardiac blood withdrawal. The success of the OVX was analyzed by body weight determination and blood plasma estrogen level determination of all mice. The bone (femur, spine) and immune phenotypes (bone marrow, spleen, inguinal lymph nodes) of all mice were analyzed via blood analyses, biomechanical testing, µCT analysis, histomorphometry, and flow cytometry, as described below.

### 4.5. Blood Analyses

For the determination of estrogen and bone turnover marker (OPG, RANKL) concentrations, plasma and serum were harvested from female mice 8 weeks after OVX or sham surgery via intracardial blood withdrawal and collected in ethylenediamine tetra-acetic acid (EDTA) microvettes or microvettes (both Sarstedt, Nümbrecht, Germany), respectively. The samples were centrifuged for serum collection at 13,000× *g* for 7 min, or for plasma collection at 10,000× *g* for 10 min. Plasma estrogen concentrations were determined by using a mouse/rat Estradiol ELISA kit (Calbiotech, El Cajon, CA, USA) according to the manufacturer’s protocol. Serum bone turnover markers were determined by using a mouse Osteoprotegerin ELISA kit (TNFRSF11B) and a mouse RANKL ELISA kit (TNFSF11) (both Abcam, Cambridge, UK) according to the manufacturer’s protocol.

### 4.6. Biomechanical Bone Testing

To analyze the biomechanical properties of the bones of female mice 8 weeks after OVX or sham surgery, femurs were harvested and kept moist in sodium chloride solution until the performance of a non-destructive three-point bending test as described previously [65]. The proximal end of the femur was mounted into an aluminum cylinder and placed in a material testing machine (Z010, Zwick, Ulm, Germany). The condyles rested unfixed on a bending support, an axial load of 4 N was applied to the femoral midshaft, and the load and deflection were recorded. The flexural rigidity of the femurs was calculated from the slope of the load–deflection curve.

### 4.7. Micro-Computed Tomography

Femurs and lumbar spines were fixed in 4% phosphate-buffered formaldehyde solution for 48 h and 14 days, respectively. µCT scanning was performed by using the Skyscan 1172 (Bruker, Kontich, Belgium) operating at 50 kV and 200 mA and with an isotopic voxel resolution of 8 µm. Analysis and calibrations were performed according to the guidelines of the American Society of Bone and Mineral Research (ASBMR) [66]. For the determination of the bone mineral density, two defined hydroxyapatite (HA) phantoms (250 and 750 mgHA/cm^3^) were scanned within each scan. The volume of interest (VOI) for cortical bone was set in the femur mid-diaphysis with a length of 168 µm and a global threshold of 641.9 mgHA/cm^3^. The VOI for the trabecular bone was defined in the distal part of the femur 200 µm proximal to the metaphyseal growth plate over a total length of 280 µm and with a global threshold of 394.8 mgHA/cm^3^. In the spine, the VOI comprised the entire trabecular region of the lumbar vertebral body L2 at a total length of 160 µm and with a global threshold of 394.8 mgHA/cm^3^.

### 4.8. Histomorphometry

Following µCT scans, femurs were subjected to decalcified histology and embedded in paraffin, while lumbar spines were subjected to calcified histology and embedded in methyl methacrylate as described previously [67]. Cellular parameters were evaluated in the femurs and lumbar vertebrae according to the ASBMR guidelines in Toluidine blue- and TRAP-stained sections (paraffin: 7 µm; methyl methacrylate: 10 µm), respectively [68]. Osteoblasts and osteoclasts were counted 100 µm proximal to the metaphyseal growth plate in the femur, and in a rectangular area (650 × 450 µm) in the middle of the trabecular part of the lumbar vertebral body L2 via light microscopy (Zeiss Axiophot, Carl Zeiss AG, Oberkochen, Germany) and by using the Osteomeasure system (v.4.1.0.0, OsteoMetrics, Decatur, GA, USA). Osteoblasts were identified in toluidine blue-stained sections by their cubic shape and direct contact to the bone surface. Osteoclasts were identified by their positive TRAP staining, two or more nuclei, and their direct contact to the bone surface.

### 4.9. Flow Cytometric Analysis

Immune cell populations in the bone marrow, spleen, and inguinal lymph nodes of female mice 8 weeks after OVX or sham surgery were determined via flow cytometric analysis. Inguinal lymph nodes and spleen were explanted and passed through a cell strainer (70 µm). The ends of the femur were removed, and bone marrow was centrifuged out (12,300 rpm for 40 s) and resuspended in phosphate-buffered saline. Lysis of erythrocytes of spleen and bone marrow samples was performed by using lysis buffer (150 nM NH_4_CL, 1 mM KHCO_3_, 0.1 mM Na_2_EDTA, all Sigma-Aldrich) for 5 min at 37 °C. Cells were stained with the following antibodies for 30 min on ice: F4/80 FITC (1:50, eBioscience, Frankfurt, Germany), Ly6G V450 (1:400, BD Bioscience, Heidelberg, Germany), CD11b Alexa Fluor 700 (1:400), CD3e PE-Xyanine7 (1:100), CD4 APC-eFluor 780 (1:200), CD8a APC (1:800), and CD19 PE (1:400, all eBioscience). Corresponding isotype controls were used. Live–dead discrimination was performed with 7-aminoactinmycin D. A BD FACSLyric^™^ flow cytometer (BD Bioscience) was used for sample measurement, and FlowJo software (10.0.8r1, FlowJo, Ashland, OR, USA) was used for analysis.

### 4.10. Statistical Analysis

Data in figures are presented as box-and-whisker plots (with median and interquartile range) from maximum to minimum, showing all data points. Data in tables are expressed as mean ± standard deviation. Depending on the experiments, we used 7–10 mice per group. Samples were allocated in a blinded manner to the different analyses, which were performed on a subset of samples because several analyses were mutually exclusive (e.g., flow cytometric analysis). The sample sizes are indicated in the figure (single data points) and table legends. Data were tested for normal distribution by using the Shapiro–Wilk normality test. Comparisons between the two groups were performed by using a two-tailed Student’s t-test in case of normal distribution or non-parametric Mann–Whitney U test if the data were not normally distributed. Comparisons between more than two groups were performed via one-way analysis of variance with post hoc Fisher’s LSD in case of normal distribution or with Kruskal–Wallis with post hoc Dunn’s test if data were not normally distributed. Values of *p* < 0.05 were considered to be statistically significant. Statistical analysis was performed by using Graph Pad Prism 9.0 software (GraphPad Software, La Jolla, CA, USA).

## 5. Conclusions

Our study implies that mast-cell-derived RANKL is not crucially involved in mast-cell-induced osteoclastogenesis or bone resorption in post-menopausal osteoporosis. Further research is needed to identify which mast cell mediators are involved in the pathology of osteoporosis.

## Figures and Tables

**Table 1:** Bone phenotype of the femur of 21–24-week-old female control and *Rankl*-KO mice.

Parameters	Control	*Rankl*-KO
CtTMD (mgHA/cm^3^)	1176 ± 68	1230 ± 34
CtTh (mm)	0.166 ± 0.002	0.158 ± 0.006 *
TMD (mgHA/cm^3^)	612 ± 49	645 ± 44
BV/TV (%)	7.4 ± 2.1	8.0 ± 2.3
TbTh (mm)	0.048 ± 0.001	0.046 ± 0.003
TbN (1/mm)	1.59 ± 0.51	1.72 ± 0.44
TbSp (mm)	0.192 ± 0.042	0.184 ± 0.03
NOb/BPm (1/mm)	13.1 ± 4.0	14.4 ± 2.6
ObS/BS (%)	8.9 ± 2.1	9.4 ± 1.2
NOc/BPm (1/mm)	6.5 ± 1.2	5.6 ± 0.5
OcS/BS (%)	11.6 ± 2.6	9.8 ± 1.5

* *p* < 0.05 compared to control. *n* = 6–9/group. HA: hydroxyapatite. CtTMD: cortical tissue mineral density. CtTh: cortical thickness. TMD: tissue mineral density. BV/TV: bone volume per tissue volume. TbTh: trabecular thickness. TbN: trabecular number. TbSp: trabecular separation. NOb/BPm: number of osteoblasts per bone perimeter. ObS/BS: osteoblast surface per bone surface. NOc/BPm: number of osteoclasts per bone perimeter. OcS/BS: osteoclast surface per bone surface.

**Table 2:** Bone phenotype of the spine of control and *Rankl*-KO sham and ovariectomized mice.

Parameters	Control		*Rankl*-KO	
	Sham	OVX	Sham	OVX
TMD (mgHA/cm^3^)	739 ± 21	693 ± 14 *	714 ± 33	684 ± 21
BV/TV (%)	24.5 ± 2.6	18.4 ± 1.3 *	24.7 ± 2.5	18.6 ± 1.6 *
TbTh (mm)	0.055 ± 0.003	0.050 ± 0.002 *	0.054 ± 0.004	0.050 ± 0.002 *
TbN (1/mm)	4.42 ± 0.33	3.69 ± 0.19 *	4.57 ± 0.43	3.75 ± 0.24 *
TbSp (mm)	0.157 ± 0.017	0.196 ± 0.024 *	0.158 ± 0.035	0.186 ± 0.035

* *p* < 0.05 compared to sham. *n* = 7/group. OVX: ovariectomy. HA: hydroxyapatite. TMD: tissue mineral density. BV/TV: bone volume per tissue volume. TbTh: trabecular thickness. TbN: trabecular number. TbSp: trabecular separation.

## Data Availability

All data are available in the main text or the Supplementary Materials and are available upon reasonable request from the corresponding author.

## Supplementary Materials

The supporting information can be downloaded at: https://www.mdpi.com/article/10.3390/ijms24119135/s1.
