# Supplementary material for: Role of Mast-Cell-Derived RANKL in Ovariectomy-Induced Bone Loss in Mice
Source: Int J Mol Sci. 2023 May 23;24(11):9135. doi: 10.3390/ijms24119135 (PMC10252757; doi:10.3390/ijms24119135)
Supplement: Supplementary file 1 [file ijms-24-09135-s001.zip › ijms-2381678-supplementary.pdf]

Supplementary Materials

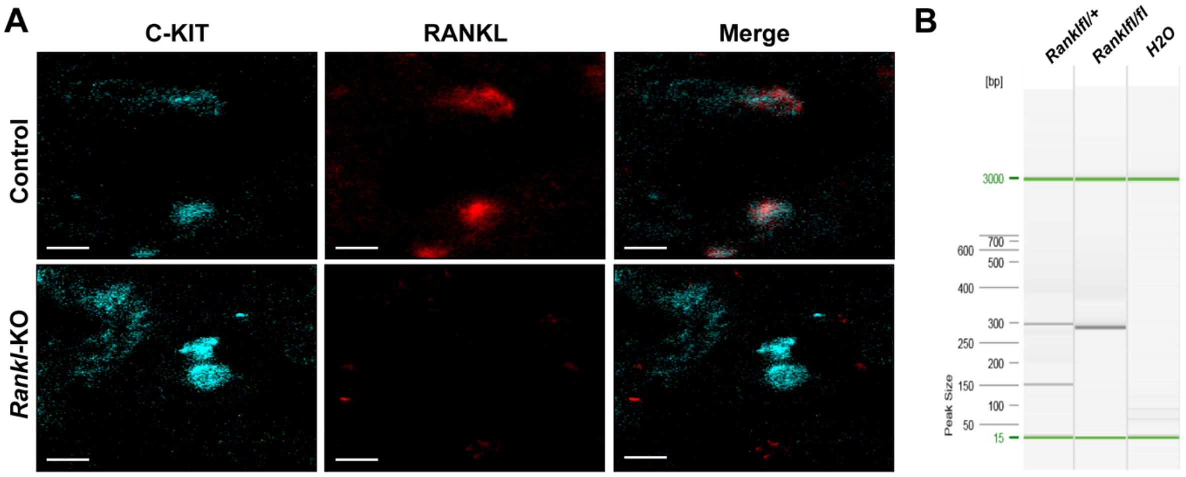

Figure S1. Specific deletion of *Rankl* in mast cells. (**a**) Immunofluorescent staining for RANKL and C-KIT (mast cells) in inguinal lymph nodes of control and *Rankl*-KO mice. (**b**) PCR-based genotyping of *Rankl*$^{fl/fl}$. Scale bar = 10 μm.

Table S1. Bone phenotype of the femur of 9–13- and 21–24–week–old male mice.

Parameters	9–13 weeks		21–24 weeks	
	Control	*Rankl*-KO	Control	*Rankl*-KO
CtTMD (mgHA/cm^3)	1225 ± 70	1213 ± 69	1265 ± 69	1236 ± 47
CtTh (mm)	0.161 ± 0.006	0.016 ± 0.006	0.166 ± 0.002	0.016 ± 0.006
TMD (mgHA/cm^3)	709 ± 69	692 ± 37	715 ± 35	713 ± 37
BV/TV (%)	12.4 ± 3.3	14.0 ± 4.7	16.7 ± 6.6	19.3 ± 3.8
TbTh (mm)	0.044 ± 0.004	0.045 ± 0.006	0.054 ± 0.008$^{\#}$	0.054 ± 0.006$^{\#}$
TbN (1/mm)	2.8 ± 0.62	3.01 ± 0.68	2.99 ± 0.92	3.55 ± 0.43
TbSp (mm)	0.137 ± 0.032	0.129 ± 0.013	0.177 ± 0.029	0.144 ± 0.026
NOb/BPm (1/mm)	11.3 ± 2.6	10.3 ± 2.6	11.4 ± 2.6	10.0 ± 1.7
ObS/BS (%)	7.8 ± 2.1	6.8 ± 1.5	8.7 ± 2.1	7.1 ± 1.2
NOc/BPm (1/mm)	6.3 ± 1.1	5.6 ± 1.1	5.7 ± 0.6	6.2 ± 0.6
OcS/BS (%)	11.0 ± 1.2	9.9 ± 1.7	9.3 ± 1.0	10.9 ± 2.2

#$p<0.05$ compared to 9–13 weeks. n=8–10/group. HA: hydroxyapatite. CtTMD: cortical tissue mineral density. CtTh: cortical thickness. TMD: tissue mineral density. BV/TV: bone volume per tissue volume. TbTh: trabecular thickness. TbN: trabecular number. TbSp: trabecular separation. NOb/BPm: number of osteoblasts per bone perimeter. ObS/BS: osteoblast surface per bone surface. NOc/BPm: number of osteoclasts per bone perimeter. OcS/BS: osteoclast surface per bone surface.

Table S2. Flow cytometric analysis of the spleen of control and *Rankl*-KO sham and ovariectomized mice.

% of living cells	Control		*Rankl*-KO	
	**sham**	**OVX**	**sham**	**OVX**
Ly6G+/CD11b+	0.93 ± 0.28	0.53 ± 0.11#	0.73 ± 0.27	0.45 ± 0.14
F4/80+/CD11b+	1.33 ± 0.23	0.97 ± 0.22	1.21 ± 0.42	1.44 ± 0.13*
CD3+	26.8 ± 3.2	25.6 ± 0.9	27.3 ± 3.0	26.0 ± 5.1
CD3+/CD4+	16.9 ± 2.1	16.0 ± 1.6	15.0 ± 1.2	16.0 ± 3.5
CD3+/CD8+	8.5 ± 1.9	7.1 ± 1.4	8.8 ± 1.3	8.4 ± 1.6
CD19+	51.1 ± 7.6	57.6 ± 4.2	50.3 ± 4.0	55.2 ± 7.4

*$p<0.05$ compared to control. #$p<0.05$ compared to 9–13 weeks. n=4–6/group. OVX: ovariectomy; neutrophils (Ly6G+/CD11b+); macrophages (F4/80+/CD11b+); T lymphocytes (CD3+); T helper cells (CD3+/CD4+); T cytotoxic cells (CD3+/CD8+); B lymphocytes (CD19+).
